# Evaluating the Performance of a Nonelectronic, Versatile Oxygenating Perfusion System across Viscosities Representative of Clinical Perfusion Solutions Used for Organ Preservation

**DOI:** 10.3390/bioengineering10010002

**Published:** 2022-12-20

**Authors:** Jose M. Gonzalez, Carorina Villarreal, Anjelyka Fasci, David Di Rocco, Sophia Salazar, Anis Khalil, Brandt Wearden, Jessica Oseghale, Mariana Garcia, Daniel J. Portillo, R. Lyle Hood

**Affiliations:** 1 Department of Mechanical Engineering, The University of Texas at San Antonio, 1 UTSA Circle, San Antonio, TX 78249, USA; 2 Department of Biomedical Engineering, The University of Texas at San Antonio, 1 UTSA Circle, San Antonio, TX 78249, USA

**Keywords:** organ preservation, kidney preservation, machine perfusion, transplantation, tissue preservation, transplantation, static cold storage, viscosity

## Abstract

**Introduction:** On the United States’ Organ Transplantation Waitlist, approximately 17 people die each day waiting for an organ. The situation continues to deteriorate as the discrepancy between harvested organs and the number of patients in need is increasing. Static cold storage is the clinical standard method for preserving a harvested organ but is associated with several drawbacks. Machine perfusion of an organ has been shown to improve preservation quality as well as preservation time over static cold storage. While there are machine perfusion devices clinically available, they are costly and limited to specific organs and preservation solutions. This study presents a versatile oxygenating perfusion system (VOPS) that supplies oxygen and pulsatile perfusion. **Materials and Methods:** Experiments evaluated the system’s performance with a human kidney mimicking hydraulic analog using multiple compressed oxygen supply pressures and aqueous solutions with viscosities ranging from 1 to 6.5 cP, which simulated viscosities of commonly used organ preservation solutions. **Results and Conclusions:** The VOPS produced mean flow rates ranging from 0.6 to 28.2 mL/min and perfusion pressures from 4.8 to 96.8 mmHg, which successfully achieved the desired perfusion parameters for human kidneys. This work provides evidence that the VOPS described herein has the versatility to perfuse organs using many of the clinically available preservation solutions.

## 1. Introduction

Transplantation of organs has increased significantly in the last few years according to the United States Department of Health and Human Services, with a 5.9% increase between 2020 and 2021, and for the first time, 40,000 organ transplants have occurred in a given year [1]. However, the deficit between the patients in need of an organ and the number who receive a transplant has continued to grow [2,3]. As an example, the kidney is the most transplanted organ worldwide, with a reported number of approximately 25,000 kidney transplants performed in the United States in 2021; yet the number of transplants performed in 2021 was approximately four-times less than the number of people on the waitlist in need of a transplant [1]. Previous studies have shown that approximately 17% of harvested kidneys are discarded due to being deemed unfit for transplantation [4]. In the United States, the organ discard rate has been approximately 9% for all organs that were harvested for transplantation [5]. Organs are discarded for reasons such as donor-related issues (virology, history, age, etc.), poor organ function, and inadequate preservation techniques (poor perfusion, extended cold ischemia times, organ damage, etc.) [6,7]. In addition, even among successful organ transplants, there remains a wide disparity in organ viability at the time of transplant that has a fundamental impact on the long-term health of the organ and recipient [8]. Therefore, organ preservation remains a critical need to ensure an organ is healthily preserved while being transported safely to the recipient’s location for transplantation.

Unfortunately, the transport process proves to be a challenging endeavor with many hurdles and considerations. Traditionally, static cold storage (SCS) at approximately 4 °C [9,10,11,12] is the clinical standard for maintaining organs during transport from donor to recipient [13,14]. SCS involves flushing donor organs to remove as much of the donor’s blood as possible and storing the organ in an ischemic (restricted or reduced blood flow) and hypothermic state. However, during the duration of the ischemic state, excessive inflammatory response from oxidative stress and other factors can result in cellular degradation [15]. Furthermore, significant organ ischemia can cause lasting effects to recipient patients. As an example, many recipients of lung transplants have demonstrated poor oxygen exchange and pulmonary infiltrates post-surgery [16]. As cold ischemia causes constant degradation, the time in which organs can be kept in this state is limited; if organs fall outside of this time range they may no longer be viable for transplantation [17]. In addition, vascular injury can occur when flow is reintroduced after a donor organ is transplanted, and together with ischemic injury, is referred to as ischemic reperfusion injury (IRI) [18]. Given these negative effects, the need for an organ preservation method that limits cellular degeneration, extends preservation times, eliminates or limits cold ischemic time, and prevents poor reperfusion is imperative for providing recipients with the healthiest organ possible while preventing organs being discarded [6].

Machine perfusion (MP) is a promising organ preservation method with the aim to eliminate or reduce the shortcomings that are associated with SCS [19]. MP involves cannulation of the major blood vessels and perfusion of a preservation solution using constant or pulsatile flow [20]. This perfusion method has been shown to improve organ viability by promoting early graft function compared to SCS [21,22]. In large animal studies, MP had better outcomes in cold preservation than SCS [23]. A study by Patel et al. demonstrated that the rate of delayed graft function was significantly less for organs preserved via MP (34.2%) compared to SCS (42.0%) [24]. Furthermore, the study identified that creatinine levels were at a more desirable concentration in patients who received organs preserved by MP as compared to SCS one-year post-transplantation. MP has also been shown to improve the health of organs that were previously deemed unfit for transplantation [25]. From these studies, the benefit of MP over SCS is becoming more widely recognized. However, current MP devices are expensive, with pricing being above USD 10,000 [26]. In addition, a subset of the devices in the preservation market lacks portability or does not supply oxygen to the organ being preserved [26]. MP devices are also limited to a specific organ and preservation solution that can be used, demonstrating a lack of flexibility and versatility [27,28].

MP systems have been developed to work with the many different solutions developed and studied for organ preservation. Solution selection has a fundamental impact on the efficacy of organ preservation as some preservation solutions benefit renal, liver, and pancreas transplants while other solutions may cause harm such as pancreatitis [29]. The University of Wisconsin preservation solution (UW) is one of the most frequently used media. It includes hydroxyethyl starch (HES), which has the benefit of inhibiting edema [30]. However, the starch has been shown to cause red blood cell aggregation in humans [31]. Another compound used in preservation solution is polyethylene glycol (PEG). The use of PEG has been shown to inhibit immune response to injury caused by kidney ischemia, improve renal function, as well as improve liver clearance and bile production [30]. However, PEG and other preservation solutions may not combat excess calcium buildup in cells, which has been shown to lead to heart cell apoptosis and necrosis [32]. Given the variety of solutions available for preservation, it is important to understand how different solutions would affect the performance in newly developed MP devices.

The versatile oxygenating perfusion system (VOPS) is a device developed by our group that aims to tackle the current shortcomings of MP devices. The VOPS device aims to be able to preserve a variety of organs with a chosen preservation solution. The VOPS device is frugal (USD 300 material cost), portable (15.2 × 39.2 cm), lightweight (6.8 kg loaded), and able to provide oxygenation to a cannulated organ [26]. A length of silicone tubing in the pump chamber provides oxygenation and perfusion circulation by cyclical inflation and deflation with compressed oxygen. The VOPS device was demonstrated to be able to reach perfusion pressures, flow rates, and oxygenation rates required to preserve a variety of organs [26]. That ability, along with the VOPS’s low cost, addresses key shortcomings of the clinically available MP systems.

Previous work has successfully shown the potential to address key shortcomings but needed to be tested with varying preservation solutions. The objective of this study was to evaluate the preservation potential of the VOPS device across a range of solution viscosities representative of the most commonly used preservation solutions in clinical care. To achieve this objective, an aqueous solution was created to mimic a range covering the previously discussed range of viscosities. Flow rates and perfusion pressures were gathered at each different viscosity with varying oxygen supply pressures.

## 2. Materials and Methods

### 2.1. VOPS Device Design

**Figure 1:**
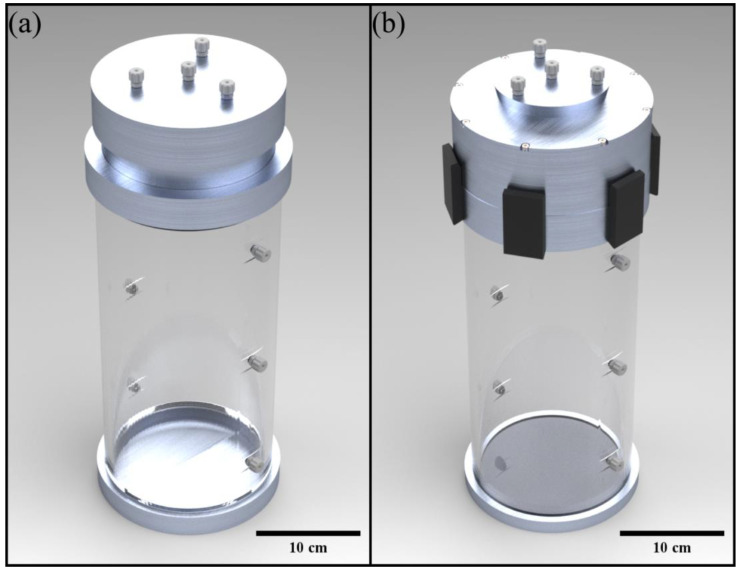
(**a**) A render of the original iteration of VOPS. (**b**) The redesigned version of the VOPS device.

The VOPS device was comprised of two primary compartments: the pump and organ chamber. The pump chamber was composed of the top cap, helical silicone tubing (Silastic^®^ Laboratory Tubing, DuPont de Nemours, Inc.), fastening lid, and resin fixture. The silicone tubing was arranged into a helix; the resin fixture was designed to prevent pinch points and maintain flow. The organ chamber consisted of two parts: the base cap and cylindrical acrylic walls. The organ chamber was machined with threaded holes on the sides to attach ports for data collection [26]. The organ storage chamber has a height of 28.5 cm and inner diameter of 12.7 cm, these dimensions were chosen to able to fit a human-sized kidney, partial living donor liver, and a heart [33,34,35]. The organ storage chamber is able to be manufactured to different sizes for inclusion of varying sized organs. A previous iteration of the VOPS device was described in Portillo et al., while the VOPS device used in this study has several improvements. These improvements included improved fittings and gaskets for achieving a better seal, simplified access to the organ chamber, and removal of threaded components in favor of latches to provide compression for the seals. The changes increase ease of access to the organ chamber while maintaining security in the pump chamber where access should be minimized for optimal performance. Additionally, a conical shape was implemented for the organ chamber and pump chamber to improve gas elimination. The top cap, pump chamber and base cap were made of 6061 aluminum, the silicon tube holder was 3-D printed in-house (Clear Resin, Form 2, Formlabs), and the organ chamber was machined from acrylic and epoxied in place with medical grade sealant. A rendering of the previous device and the current device are shown in Figure 1.

### 2.2. VOPS Device Operation and Experimental Setup

**Figure 2:**
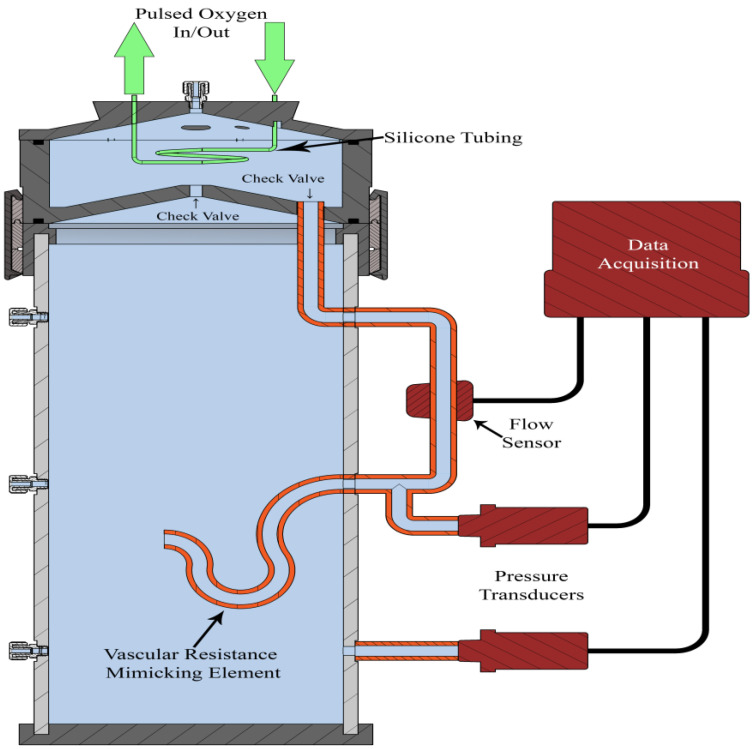
Section view of the redesigned VOPS experimental setup.

The VOPS device operates using pulsatile, pressurized oxygen to cyclically inflate and deflate silicone tubing immersed in a preservation solution. This enables both oxygenation of the solution through the permeable silicone and pumping of the solution through the rest of the system. As the pressurized oxygen entered the tubes (inflation), the pressure within the pump chamber increased above the hydrostatic pressure in the main organ chamber. This pressure variance forced a bolus of oxygenated fluid from the pump chamber into the main organ chamber through a perfusion port [26]. As the pressure dropped, the silicone tubing deflated, subsequently dropping the pressure in the pump chamber beyond the pressure in the main organ chamber. This caused a bolus of oxygenated fluid to return to the pump chamber; this process allowed for a pulsatile flow throughout the device. The silicone tubing contained nanopores in its microstructure that prevented free liquid molecules from entering the silicone tubing but allowed oxygen transport [36]. Thus, the pressurized oxygen used in the system diffused into the solution used in the VOPS device. Specified lengths of flexible polyvinyl chloride (PVC) tubing were used to mimic the vascular resistance of a kidney. Pressure transducers (PX309-030G5V, Omega Engineering, Inc., Norwalk, Connecticut, USA) were attached to the inlet of the vascular resistance-mimicking PVC element and the main organ chamber; the measured difference between the pressures at these tubes indicated the perfusion pressure. In addition to the perfusion pressure, the flow rate was measured by a sensor (2PXL-TS410, Transonic Systems Inc., Ithaca, New York, USA) attached to a flow loop located outside of the device. The pressure transducer and flow sensor data was recorded via a LabVIEW virtual interface (NI 9221 and NI USB-9162, LabVIEW 2019 SP1, National Instruments, Austin, Texas, USA) and analyzed in MATLAB (MATLAB R2020a, The MathWorks, Inc., Natick, MA, USA). The entire VOPS experimental setup is visualized in Figure 2.

### 2.3. Viscosity Theory and Criterion Determination

The VOPS was previously studied using a single fluid, phosphate-buffered saline (PBS) [26]. A prominent property of fluids, that often dictates how a fluid will flow within a fluid system, is viscosity [37]. The dynamic viscosity of the fluid, μ, or just viscosity, is often what is thought of when viscosity is referenced. Kinematic viscosity, also known as the momentum diffusivity, is a ratio of the dynamic viscosity (μ) of a fluid over the density of the fluid (ρ), shown in Equation (1).

ν = μ ÷ ρ,(1)

When measuring viscosity, the kinematic viscosity is often the output of certain viscometers, thus requiring density to convert it to a dynamic viscosity for ease of use. Viscosity measurements are also affected by a variety of factors, such as the temperature of the liquid, the pressure of the gas, and low-pressure gasses are affected by temperature, and accuracy of measurements are poor near critical points of fluids [38]. At room temperature, the dominant force on viscosity in a liquid is temperature, thus pressure effect on viscosity was able to be neglected.

VOPS characterization based on viscosity was important due to the variety of organ preservation solutions used clinically. Table 1 exhibits several preservation solutions and their viscosities identified in the literature [39,40,41,42,43,44,45,46]. Preservation solutions were removed from consideration if they were no longer being manufactured or if no viscosity data were available.

To encompass the viscosities of the preservation solutions found in the literature, a range of viscosities between 1–6.5 cP were chosen to be tested. An aqueous calcium chloride solution was identified as being Newtonian and capable of attaining the range of viscosities desired [47]. Calcium chloride dihydrate (Flinn Scientific, Inc, Batavia, IL, USA) was mixed at mass percentages of 1, 2, 3, 4, 5, 10, 15, 20, 30, and 40%. A Cannon-Fenske Viscometer (CANNON Instrument Company, State College, PA, USA) was used to find the viscosity of the aqueous solutions with the varying mass percentage. The fluid was allowed to reach temperature equilibrium inside the viscometer. Five tests were performed at each mass percentage and the resulting viscosity data were output in centiStokes. The average of these 5 tests was obtained as the viscosity at the respective mass percentage. The density of the solution was gathered at each experiment set by measuring the mass of the calcium chloride dihydrate and the volume of the fluid. Using these measured values as well as the density of water (ρ = 0.998 g/mL) and density of calcium chloride dihydrate (ρ = 1.85 g/mL) at 20 °C, the density was found using the Equation (2).

ρ_solution_ = (m_CaCl_2_ dihydrate_ + m_water_) ÷ (V_CaCl_2_ dihydrate_ + V_water_), (2)

The product of the water’s volume and the density of the water results in the mass of the water (m_water_) and the value of the calcium chloride dihydrate’s mass divided by the density is the volume of the calcium chloride dihydrate (V_CaCl_2_ dihydrate_). Multiplying the measured viscosities in centiStoke with the density at each respective mass percentage achieved unit consistency with the viscosities found in the literature by converting everything to dynamic viscosities. Mass percentage values were found at each viscosity by a 5th order polynomial curve that tracked the plotted viscosity values that were measured with a high degree of accuracy (polynomial fits of various orders are shown in Table A1).

Viscosities to be tested are shown in Table 2. With the determined viscosities, the vascular resistance element lengths were able to be calculated for the experimental setup. The lengths of these vascular resistance elements had to be found for each viscosity as they varied with the value of viscosity. The kidney’s vascular resistance was used in the Hagen-Poiseuille equations to find the appropriate length of the PVC element [48], by plugging Equation (3) into (4) and solving for length resulting in Equation (5).

Q = Δp × (πr^4^) ÷ (8μL), (3)

Δp = Q × R,(4)

L = (Rπr^4^) ÷ 8μ, (5)

where Q is flow rate, ∆p is the pressure difference, r is radius, μ is viscosity, L is length, and R is vascular resistance. The kidney mimicking vascular resistance had an inner radius of 0.75 mm. The lengths of these PVC tubes were calculated to be 27.5, 9.1, 5.5, and 4.2 cm at 1, 3, 5, and 6.5 cP, respectively.

The range of viscosities being tested indirectly covers the impact of temperature on the device’s performance. The range of values from the literature includes solutions such as a solution with a viscosity of 6.2 at 1 °C. While the VOPS has not been tested at 1 °C, the fluid will behave as it is at 1 °C due to the viscosity.

### 2.4. Determining Tuned Pulse Rates

Similar methods were used to tune the pulse rates as Portillo et al. [26], with the aim to increase the perfusion flow rates produced by VOPS. In brief, pulse profiles of each trial (*n* = 50) were averaged together to create an average pulse profile. The ideal time for an oxygen delivery valve to be closed was determined to be when the pressure in the organ storage chamber returned to 2% of the initial pressure [26]. To maximize the flow rate, the ideal amount of time for oxygen to be delivered was needed. This was found by integrating the average flow profile of the trial, with the ideal closed time accounted for. The combination of these two times provided the tuned pulse frequency by being the open/close time (also on/off). These tuned rates were used at the varying viscosities and oxygen pressures.

The tuned timings were implemented via a pneumatic circuit (Clippard) as described in another study by Portillo et al. [49]. Briefly, the current study utilized a VOPS configuration without any electronic control, but instead leveraged a pneumatic circuit tuned with manual dials to achieve desired on/off times. A 20 second test trial was conducted, gathering pressure data of the VOPS device. A MATLAB script was written to determine a maximum and minimum pressure of the test trial which were then averaged to create a threshold pressure value. The script determined when the pressure readings were above or below the threshold value and output a square waveform reflecting those values. The time location when the square wave is at a maximum or minimum was used to calculate the average on and off time of the test trial [49]. These test trials were conducted to achieve the 2 second on, 2 second off baseline trials as well as the timing required in the tuned trials.

### 2.5. Test Matrix for VOPS Characterization

To characterize the performance of the VOPS platform, fluid parameters were varied in experimental trials, which included solution viscosity (cP), oxygen pressure (kPa), and pulse rate (Hz). The temperature of the solution remained constant throughout all trials (20 °C ± 1 °C). The viscosities of various concentrations of aqueous calcium chloride within the device included 1, 3, 5, and 6.5 cP. A constant vascular resistance of 0.22 mmHg/mL/min was used to simulate the typical hydraulic resistance measured in human kidneys [24]. Oxygen pressures assessed included 27.6, 55.2, 82.7, and 110.3 kPa, which were introduced into the device top chamber via tuned pulse rates. The silicon tube length housed within the top chamber was held constant throughout the test matrix as well to determine the variable impact of viscosity to the device peak perfusion pressure, mean flow rate, and oxygenation rate during each trial. A silicone tube 6.1 m in length was used for each trial as previous work showed this length produced the widest range of acceptable perfusion pressures and flow rates [26,49]. The testing matrix is shown in Table 3.

## 3. Results

### 3.1. Current Device Performance

The current VOPS device, due to changes in the pump chamber geometry, had different mean flow rates and peak perfusion pressures than the previous iteration of VOPS. The changes in performance were compared with the first iteration of VOPS at equivalent experimental configuration. The silicone tubing length was approximately 6.1 m in both setups, PBS was used as the working fluid, and the experiments were both at 20 °C. Both experimental setups were carried out at four varying oxygen supply pressures. The performance differences are presented in Table 4.

The current iteration of the VOPS device had mean flow rates that were between 28.8 to 46.8% greater than the performance of the previous iteration of VOPS. The current iteration had mean flow rates between 3.5–11.8 mL/min. The peak perfusion pressures of the current device were between 16.1 and 52.2 percent greater than the previous VOPS device. The peak perfusion pressure of the current device was between 37.2–89.9 mmHg

### 3.2. Calcium Chloride Dihydrate Viscosity Range

**Figure 3:**
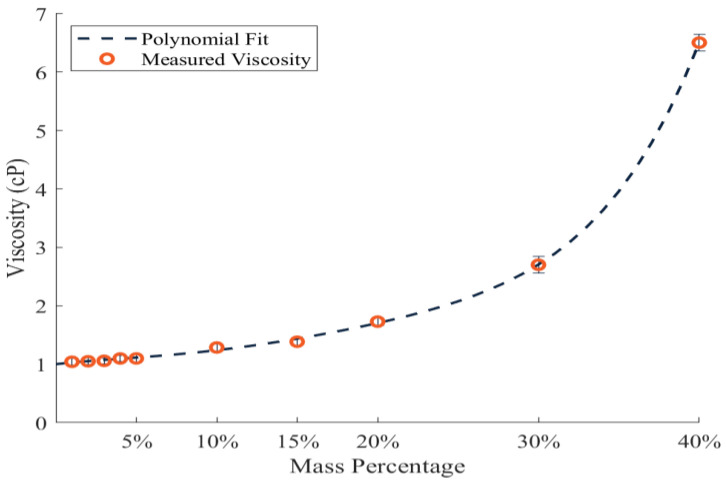
Viscosity of CaCl_2_ Dihydrate (Data markers at lower mass percentages exceed the size of the error bars).

Calcium chloride dihydrate was mixed at mass percentage ratios ranging from 1% to 40%. The range of viscosities measured successfully covers the range of viscosities necessary to cover the range of preservation solution viscosities presented (1.00–6.20 cP) and are presented in Figure 3.

### 3.3. Current Device Performance

#### 3.3.1. Baseline Data

**Figure 4:**
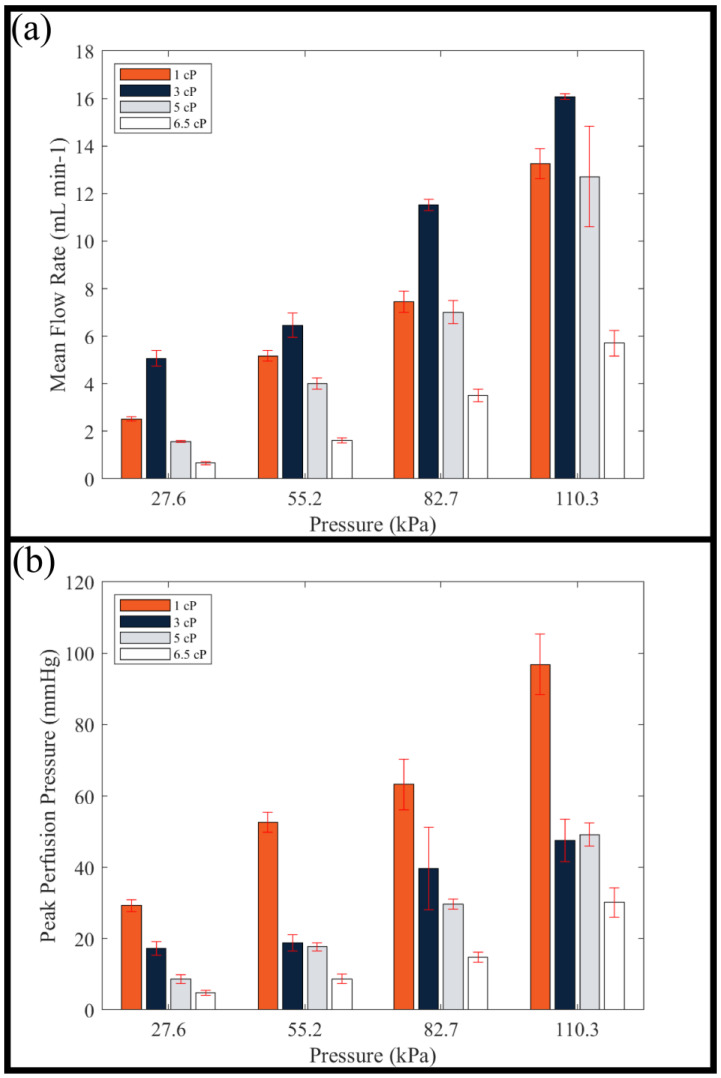
(**a**) Mean flow rate trend with increasing viscosity at each oxygen supply pressure. (**b**) Peak perfusion pressure with increasing viscosity at each oxygen supply pressure.

The baseline data was gathered to determine the effect of viscosity on the pulsatile perfusion pressure and flow rate through a consistent vascular resistance and silicone tube length in the pump chamber. The effect that the viscosity of the solution had at four varying oxygen supply pressures, with all other variables equal, are presented in Figure 4.

At a viscosity of 1 cP, mean flow rates ranged between 2.5 ± 0.10 to 13.2 ± 0.63 mL/min at oxygen supply pressures between 27.6 to 110.3 kPa. Peak perfusion pressures varied between 29.3 ± 1.67 and 96.8 ± 8.42 mmHg. At equal oxygen supply pressures, the 3 cP viscosity had mean flow rates between 5.0 ± 0.33 and 16.0 ± 0.11 mL/min and peak perfusion pressures between 17.2 ± 1.98 and 47.5 ± 6.01 mmHg. A viscosity of 5 cP produced mean flow rates and peak perfusion pressures between 1.5 ± 0.04 to 12.7 ± 2.12 mL/min and 8.6 ± 1.16 to 49.2 ± 3.25 mmHg, respectively. The highest viscosity tested, 6.5 cP, had mean flow rates between 0.6 ± 0.07 and 5.7 ± 0.54 mL/min and peak perfusion pressures between 4.8 ± 0.74 and 30.1 ± 4.08 mmHg. Overall, as viscosity increased, the peak perfusion pressures experienced by VOPS decreased at all different oxygen supply pressures. As viscosity increased, the mean flow rates initially increased between 1 and 3 cP, but then saw a decrease between 3 and 6.5 cP.

#### 3.3.2. Data with Tuned Parameters

**Figure 5:**
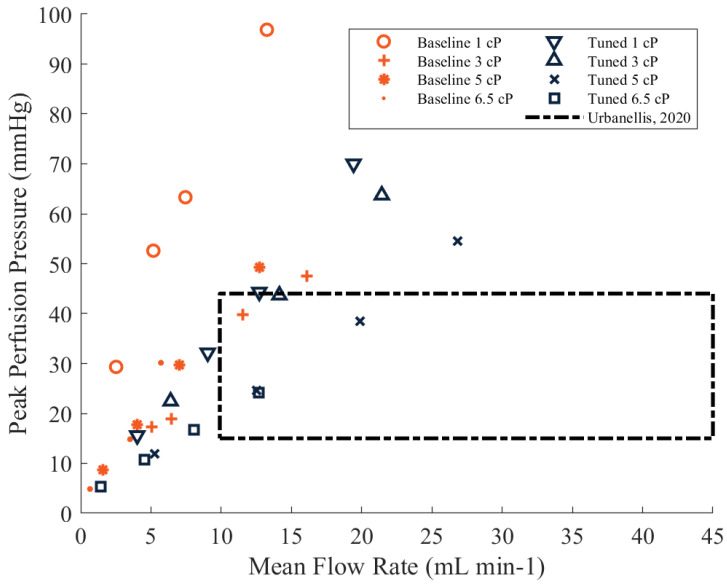
Peak Perfusion Pressure and Mean Flow Rate produced by VOPS (vascular resistance of 0.22 mmHg/mL/min). Box represents the range of values utilized in previous preservation studies [50].

Peak perfusion pressures and mean flow rates that were gathered during the baseline trials and the tuned data trials are shown in Figure 5. Each respective data point is a mean value (*n* = 5) of the pump configuration of a 20 ft silicone tube with a vascular resistance of 0.22 mmHg/mL/min. In addition to the mean flow rates and peak perfusion pressures, Figure 5 displays the top range and bottom range of peak perfusion pressures and mean flow rates used by previous groups for the preservation of a kidney. The minimum and maximum of the peak perfusion pressures are represented by the bottom and top of the box, respectively, and the minimum and maximum of the mean flow rates are represented by the left and right side of the box, respectively.

Tuning the pulse frequencies and duty cycles used in the VOPS experimentation tended to decrease the peak perfusion pressures and increase the mean flow rates. At a viscosity of 1 cP, mean flow rates ranged from 4.0 ± 0.11 to 19.4 ± 0.42 mL/min and peak perfusion pressures ranged between 15.5 ± 1.06 to 70.0 ± 4.89 mmHg. A viscosity of 3 cP used in the VOPS device produced mean flow rates between 6.4 ± 0.06 and 28.2 ± 0.26 mL/min alongside a range of peak perfusion pressures between 22.4 ± 1.19 to 83.8 ± 1.05 mmHg. The VOPS device, at a viscosity of 5 cP, produced mean flow rates between 5.2 ± 0.07 and 26.8 ± 0.24 mL/min and peak perfusion pressures between 11.8 ± 0.96 and 54.5 ± 1.17 mmHg. At the highest viscosity of 6.5 cP, VOPS produced flow rates between 1.4 ± 0.23 and 12.6 ± 0.10 mL/min and peak perfusion pressures ranging from 5.3 ± 0.67 to 24.2 ± 1.30 mmHg. The results from the kidney specific vascular resistance established the VOPS device’s ability to supply mean flow rates and peak perfusion pressures within the target ranges obtained in previous porcine kidney preservation studies conducted by Urbanellis et al. at all viscosities tested [50].

#### 3.3.3. Study Limitations

Vascular resistances represented in this study are limited to one value, when the same organ in a group of individuals may have unique vascular resistances. The vascular resistance element also lacks the compliance that the vasculature in an organ would have, likely resulting in a different behavior as the PVC element in this study did not contract and expand. The vasculature resistance of an organ will also vary with temperature and the preservation time. Using a vascular resistance mimicking element with compliance as well as broadening the range of vascular resistance values tested on the VOPS device would further improve the study and the understanding of how the VOPS device could potentially aid in organ preservation.

## 4. Discussion

The VOPS device was updated to better improve sealing, simplify access to the organ chamber, and improve the geometry of the pump chamber. These changes were significant, requiring a comparison in peak perfusion pressures and mean flow rates to the previous iteration of the VOPS device. This test was performed to see how the changes, primarily the sealing and pump chamber improvements, would affect the mean flow rates and peak perfusion pressures. All variables were held constant between this test and testing of the previous iteration of the VOPS device, aside from the described changes in design. When compared to the previous iteration of the VOPS device, the updated model had an increase in performance at all oxygen supply pressures. Mean flow rates increased between 28.8 and 45.7% over the previous iteration of the VOPS device and peak perfusion pressures increased between 16.1 and 52.2% [49]. The redesigned VOPS device produced mean flow rates between 3.5 and 11.8 mL/min and peak perfusion pressures between 37.2 and 89.9 mmHg.

The calcium chloride dihydrate aqueous solution was chosen as a mimic for clinically used preservation solutions as it is Newtonian and mixtures at different concentrations were able to capture the range of viscosities exhibited by those solutions [51,52,53,54,55,56,57]. The latter was validated through viscometry, demonstrating the 1–40% aqueous calcium chloride solution could span 1–6.5 cP. The viscosities measured followed a similar trend to pure calcium chloride aqueous solutions found in the literature, which validated the exponential behavior documented [58].

The VOPS device successfully reached the perfusion parameters needed to preserve a kidney [50]. Using a silicone tube length of 6.1 m, all viscosities had an oxygen supply pressure that met the necessary parameters. A viscosity of 1 cP had produced desirable perfusion parameters at a pressure of 82.7 kPa. At 3 cP, the VOPS device had perfusion parameters desired at 55.2 kPa. At a viscosity of 5 cP, supply pressures of 55.2 and 82.7 kPa produced mean flow rates and perfusion pressures that fell in the range of perfusion parameters for a kidney. At the highest viscosity of 6.5 cP, a supply oxygen pressure of 110.3 kPa produced perfusion parameters that were desired. Previous work by other groups has demonstrated the benefits of MP [20,21,24,28,30,50,59,60,61,62,63]. The VOPS device being able to reach target perfusion parameters at varying viscosities indicates that the VOPS device may be able to improve the viability of organs transplanted, the quality of the transplanted organs, as well as improving the organ preservation time with a variety of different preservation solutions of varying fluid viscosities.

Future alterations can include geometric changes to the pump chamber, altering the dimensions of the silicone tubing, as well as altering the length and quantity of the silicone tubing used in the pump chamber. The VOPS device will also need to be tested at hypothermic and normothermic temperatures, as it has been previously demonstrated that some organs benefit from preservation at different temperature ranges [64]. Ex vivo studies are planned and will test the preservation capabilities of the VOPS device for a variety of solid organ grafts, such as kidneys, hearts, and livers. The process of simulating a computational model of the VOPS device is work that is being planned. The purpose of performing simulation tests is to predict how various variable configurations might affect the results and to reduce physical benchtop testing.

## 5. Conclusions

The VOPS device has benefits over current organ preservation devices due to its portability, versatility, low cost, and ability to be operated either electrically or non-electrically. The experiments performed show an ideal viscosity that would be preferable for the VOPS device where most of the oxygen supply pressures tested reached desirable perfusion parameters for the preservation of a human kidney. While the VOPS device met the perfusion parameters at more oxygen supply pressures with one tested viscosity, all viscosity solutions tested reached the flow parameters necessary for kidney preservation. This indicates that VOPS is highly versatile as it can potentially be used with several preservation solutions to suit clinical need.

## Figures and Tables

**Table 1:** Viscosity Range of Preservation Solutions.

Preservation Solution	Viscosity	Temperature
PBS	1.0 cP [39]	25 °C
Celsior	1.3 cP [40]	5 °C
IGL-2	1.4, 1.7 cP [41,42]	4 °C, N/A
STEEN	1.5, 4.5 cP [43]	37, 4 °C
HTK	1.8, 2.0 cP [40,44]	5 °C
Polysol	1.8 cP [40]	5 °C
BMPS	2.4, 2.6 cP [41,42]	4 °C, N/A
Blood *	3.5–5.0 cP [45]	N/A
ET-K	4.0 cP [46]	4.5 °C
UW	5.5, 5.7, 6.2 cP [40,44,46]	4.5, 5, 1·°C

* Included for reference.

**Table 2:** Determined Mass Percentages.

Viscosity Desired	Mass Percentage Required
1 cP	0%
3 cP	31.53%
5 cP	37.47%
6.5 cP	39.99%

**Table 3:** Testing Matrix.

Length of Tubing	Vascular Resistance	Oxygen Pressure	Viscosity
6.1 m	0.22 mmHg/mL/min	27.6 kPa	1 cP
		55.2 kPa	3 cP
		82.7 kPa	5 cP
		110.3 kPa	6.5 cP

**Table 4:** Current Device Performance.

	Mean Flow Rate (mL/min)	Difference	Peak Perfusion Pressure (mmHg)	Difference
27.6 kPa	3.5	45.7% ↑	37.2	52.2% ↑
55.2 kPa	6.8	44.1% ↑	59.1	42.8% ↑
82.7 kPa	10.9	46.8% ↑	86.2	45.6% ↑
110.3 kPa	11.8	28.8% ↑	89.9	16.1% ↑

## Data Availability

The datasets generated during and/or analyzed during the current study are available from the corresponding author upon reasonable request.

## Appendix A

**Table A1:** Polynomial Fits of Viscosity Data.

	1st Order	2nd Order	3rd Order	4th Order	5th Order
b_5_					2373.786
b_4_				698.1803	−1561.11
b_3_			215.5589	−347.005	394.9193
b_2_		52.69206	−76.6822	65.46186	−33.4869
b_1_	11.27494	−8.927	10.24144	−1.74879	3.106763
b_0_	0.429868	1.32782	0.84621	1.063192	1.001567
R Square	0.7783	0.9693	0.9971	0.9996	0.9998
RSME	0.8483	0.3377	0.112	0.0432	0.039
